# Pakistan Following Foot Prints of Developed World in Structural Interventions: Experience of Transcatheter Aortic Valve Implantation Reported First Time

**DOI:** 10.7759/cureus.11497

**Published:** 2020-11-16

**Authors:** Ali Ammar, Syed N Hassan Rizvi, Tahir Saghir, Naveedullah Khan, Parveen Akhtar, Naeem Mengal, Jawaid A Sial, Nadeem Qamar

**Affiliations:** 1 Adult Cardiology, National Institute of Cardiovascular Diseases, Karachi, PAK; 2 Interventional Cardiology, National Institute of Cardiovascular Diseases, Karachi, PAK; 3 Cardiology, National Institute of Cardiovascular Diseases, Karachi, PAK; 4 Cardiology/Interventional Cardiology, National Institute of Cardiovascular Diseases, Karachi, PAK

**Keywords:** transcatheter aortic valve replacement, bicuspid aortic valve, atrioventricular block, aortic valve stenosis, heart valve diseases

## Abstract

Background

The aim of this study was to evaluate the safety and efficacy of transcatheter aortic valve implantation (TAVI) program in a Tertiary care hospital in Karachi, Pakistan.

Methodology

This study was conducted by interventional cardiology department of the National Institute of Cardiovascular Diseases (NICVD), Karachi from July 2015 to February 2020. All patients of severe aortic stenosis (AS) who underwent TAVI were included. Baseline characteristics, in-hospital course and one-month follow-up data were collected.

Results

This study included 100 consecutive patients with severe AS undergoing TAVI. Sixty-three (63.0%) patients were males and the mean age was 67.38 ± 10.73 years. Eighty-five (85%) patients were in the New York Heart Association (NYHA) class III-IV. Aortic valve mean gradient was 51.33±10.47 mmHg and 50% of patients had bicuspid aortic valves. Core valve was implanted in 86 (86%) and evolute-R aortic valve was implanted in 14 (14%) patients. TAVI was successfully done in 94% of patients. Post-deployment aortic valve mean gradient was 5.33±4.13 mmHg. Major vascular access site complications were noted in 14% and atrioventricular (AV) blocks were seen in 22% of cases. There was a significant difference in symptoms of patients before and after the procedure. Overall, eight (8%) patients expired during hospital stay. At one-month follow-up, 76% of patients were found to have no limitation of physical activities.

Conclusions

Results of this study showed that TAVI is a safe procedure in these high-risk patients and is an alternative to surgery for AS patients in the region.

## Introduction

Aortic stenosis (AS) is the disease of the elderly and its prevalence increases with increasing age [1]. In untreated patients, it is suggested that the valve area decreases by 0.1 cm^2^ per year [2]. Once the symptoms start, the prognosis of untreated patients becomes poor. In symptomatic AS patients, the estimated annual mortality rate is 25% [3], which is quite high. Surgical aortic valve replacement (SAVR) remained the gold standard in the treatment of severe AS patients [4]. But because of advanced age, frailty and comorbid conditions, many patients are considered high risk for SAVR. For such patients, for the first time, transcatheter aortic valve implantation (TAVI) was done in 2002 by Allen Crebier in France. Since then TAVI became the treatment of choice for inoperable and high-risk severe AS patients [5].

Osnabrugge et al. in their study showed that 40% of all severe symptomatic AS patients were unable to go for surgery because of age and associated risk factors [6]. Out of them, 5.2% were TAVI candidates. They projected that in European countries, there are approximately 189,836 TAVI candidates while in North America there are approximately 102,558 candidates. According to Asteggiano et al. free access to TAVI in Asian/ Oceania countries is 20% while in Africa it is 2.4% [7]. Pakistan is a developing country in which the National Institute of Cardiovascular Diseases (NICVD) is the first institute to develop TAVI program not only for its own population but also for the surrounding region.

In Asia, Singapore started the TAVI program in 2009. In the Middle East and nearby areas, Turkey was the first country to start TAVI program [8] followed by Iran and Saudi Arabia in 2010 [9,10]. In developing countries, cost-effective treatment is important and in high-risk patients, SAVR is still cost-effective. However, in inoperable patients, TAVI is the only choice. With recent trials; PARTENAR III trial and Evolute low-risk trial, TAVI now became cost-effective in low and intermediate-risk patients and even it became equally safe and effective as SAVR [11]. Moreover, data showed that in TAVI ICU stay was significantly shorter as compared to SAVR (three days vs. five days, p< 0.001). Subsequently, the duration of hospitalization was also lower in TAVI group as compared to SAVR group (eight days vs. 12 days, p< 0.001) [12]. Since TAVI involve high-risk patients, so all cause 30 days, one year and three years observed survival estimates are lower, which are 94.7%, 83.4% and 64.5%, respectively [13].

Performing TAVI requires not only technical expertise but also carries high procedural and equipment cost. Because of these two reasons, TAVI is mainly performed in developed countries. In developing countries, TAVI is still in the adapting phase. In Pakistan, NICVD is the first public sector hospital which has developed a structured TAVI program. After initial few procedures which were done under the supervision of proctor, now the institute is running TAVI program by itself. The aim of this study was to monitor and evaluate treatment outcomes, safety and quality of this uprising, innovative and expensive method of treatment in this public sector institute. This is the landmark study that will help in establishment of safety and efficacy of this new procedure in treatment for high-risk patients of AS in our country. This new procedure will help in creating hope for patients who were otherwise left symptomatic despite medical management.

## Materials and methods

This prospective cohort study was conducted by the Department of Interventional Cardiology of NICVD, Karachi. Duration of study was from July 2015 till February 2020. Data were collected on comprehensive questionnaire which included different parameters. All variables were recorded during the index hospitalization. Variables included were history, baseline labs, echocardiography (echo), cardiac catheterization, TAVI procedure, post-procedural complications, and one month of follow-up. Ethical review committee approval was taken. This study included all patients of severe symptomatic AS, who were selected for TAVI after a series of baseline investigations. Investigations started from detailed history, examination and transthoracic echo. All TAVI patients were analyzed with electrocardiography (ECG) gated Multi-detector computed tomography (MDCT) using 3 Mensio structural heart software (Pie Medical Imaging, Maastricht, the Netherlands). Trans-esophageal echocardiography was done before, during and after the procedure. Post-procedure computed tomography (CT) scan was done only if clinically indicated. Patients were classified into high, moderate and low-risk categories according to the Society of Thoracic Surgeons (STS) and EURO II scores. Final selection of patients was done by heart team approach. Patients of severe symptomatic AS with moderate to high surgical risk for surgical aortic valve replacement fulfilling all anatomical and physical criteria for TAVI, were included in the study. Few low-risk (STS and EURO II score) patients who were inoperable or had comorbids were selected for TAVI after heart team discussion. Patients who were asymptomatic or were with moderate AS or were not fulfilling the criteria for TAVI were rejected. Written consent was taken from all the patients undergoing the procedure. At the end of the procedure, transthoracic echocardiography (TTE) was done in all patients to observe post procedure valve parameters and complications. The majority (96%) of TAVI procedures were done electively under general anesthesia, while few (4%) in conscious sedation using standard technique. The transcatheter heart valve used was Corevalve & Evolute R (Medtronic, Inc; Minneapolis, MN). In-hospital course of these patients were monitored for any complications. One-month follow-up was done and symptoms were assessed.

Data that were collected on a pre-designed questionnaire were entered using the Census and Survey Processing System (CSPro) 7.0. It was analyzed in Statistical Package for the Social Sciences (SPSS) version 21 (IBM Corp., Armonk, NY). Mean ± standard deviation (SD) were calculated for continuous variables and frequency and percentages were calculated for categorical variables. No missing data imputation technique was adopted during the analysis.

## Results

This study included 100 patients who underwent TAVI. Out of 100 patients, 63 (63.0%) were males. The mean age of patients was 67.38 ± 10.73 years. Their basic demographics are shown in Table 1.

**Table 1:** Medical history, risk factors for coronary disease, and baseline echocardiographic assessment MI, myocardial infarction; COPD, chronic obstructive pulmonary disease; TIA, transient ischemic attack; STS, society of thoracic surgeons

Characteristics	Overall
	(n=100)
Co-morbids	
Diabetes	51 [51%]
Smoking Status	
Ex-smoker	24 [24%]
Current smoker	11 [11%]
Creatinine	1.29 ± 0.67
Clinical history	
Prior MI	29 [29%]
History of Asthma/ COPD	23 [23%]
Chronic liver disease	12 [12%]
Prior Stroke or TIA	14 [14%]
Carotid or Peripheral Arterial Disease	2 [2%]
Poor mobility because of Joint Issues	14 [14%]
STS Score based risk categorization	
High risk (>8)	39 (39%)
Moderate risk (4-8)	55 (55%)
Low risk (<4)	6 (6%)
EURO-II Score based risk categorization	
High risk (>5)	36 (36%)
Moderate risk (2-5)	61 (61%)
Low risk (<2)	3 (3%)
Nature of Aortic Valve	
Tricuspid Aortic Valve	50 (50%)
Bicuspid Aortic Valve	50 (50%)
Pre-procedure echocardiographic parameters	
Aortic valve area (cm^2^)	0.82 ± 0.18
Aortic valve mean gradient (mmHg)	51.33 ± 10.47
Aortic valve peak gradient (mmHg)	81.48 ± 15.29
Aortic annular diameter (mm)	24.76 ± 3.08
Pulmonary artery systolic pressure (mmHg)	37.47 ± 29.89
Types of Implanted Valves	
1st generation Core Valve	86 (86%)
2nd generation Core Valve (Evolute R)	14 (14%)
Valve size (mm)	
23 mm	6 [6%]
26 mm	45 [45%]
29 mm	28 [28%]
31 mm	10 [10%]
34 mm	11 [11%]

All patients included in the study were symptomatic. After the procedure, significant improvement was seen in NHYA class from NYHA III-IV to NYHA I-II as shown in Table 2. During pre-procedure echo assessment, left ventricular function was normal in 48%, mild to moderate dysfunction in 30%, and severe dysfunction in 22% of patients. Other valve parameters are shown in Table 2.

**Table 2:** Pre- and post-TAVI echo parameters TAVI, transcatheter aortic valve implantation; AV, aortic valve; NYHA, New York Heart Association

	Pre-TAVI	Post- TAVI*	P-value
Valve Parameters			
AV Mean Gradients (mmHg)	51.33 ± 10.47	5.33 ± 4.13	<0.001
AV Peak Gradients (mmHg)	81.48 ± 15.29	8.84 ± 4	<0.001
AV Area (cm^2^)	0.82 ± 0.18	2.75 ± 1.02	<0.001
New York Heart Association (NYHA) Functional Classification			
NYHA I, II	11 [11%]	95 [95%]	<0.001
NYHA III	69 [69%]	1 [1%]	
NYHA IV	16 [16%]	0 [0%]	
Not assessed/missing	4 [4%]	4 [4%]	

Average procedural time was 91.04 ± 28.92 minutes. In all cases, access site was closed via device, the Proglide. First- and second-generation CORE valves were implanted in these patients. CORE valve of 26 mm was the most frequently used valve (Table 1).

Valve was successfully deployed in 94% of cases. Valve mal-positioning occurred in nine patients, which was successfully tackled by bail out valve in valve during procedure. Post implantation balloon dilatation was done where needed.

During and after the procedure, arrhythmia was the main complication (in 31 cases). Main arrhythmias were atrioventricular (AV) blocks followed by supraventricular tachycardia (SVT). Temporary pacemaker was implanted in all patients for rapid pacing and for arrhythmias. Permanent Pacemaker was implanted in 22 patients. Vascular access site bleeding and access related complications were observed in 27 (27%) patients (14% major and 13% minor). Average number of units of blood transfused during hospitalization were 1.94 ± 2.58. Out of 100 patients, eight patients (8%) expired during or post-procedure during hospitalization as shown in Table 3.

**Table 3:** Procedural complications and in-hospital outcomes TAVI, transcatheter aortic valve implantation; CVA, cerebrovascular accident; AV, atrioventricular; GI, gastrointestinal. *As per the Valve Academic Research Consortium-2 classification.

Characteristics	Overall (n=100)
Successful Valve deployment	94 [94%]
CVA up to discharge	4 [4%]
Vascular access site and access related complications	
*Major	14 [14%]
*Minor	13 [13%]
Percutaneous closure device failure	13 [13%]
Acute Kidney Injury within 7 days of procedure	
Stage 1	14 [14%]
Stage 2	6 [6%]
Stage 3	2 [2%]
Cardiac Tamponade during/post-procedure	
Requiring surgical intervention	2 [2%]
Requiring percutaneous intervention	3 [3%]
Conversion to full sternotomy during procedure for any reason	3 [3%]
Persistent AV Block	22 [22%]
Sepsis	8 [8%]
GI Bleeding	1 [1%]
Re-Intubation	9 [9%]
Mortality	8 [8%]
TAVI related mortality	4 [4%]
Mortality because of other causes (CVA/sepsis)	4 [4%]

After procedure, 74% of patients were extubated within the first 24 hours while the rest were extubated after one day. In three cases, procedure was done under conscious sedation because of high co-morbidities. Post-procedure, moderate aortic regurgitation (AR) was found in 12 patients (12%), mild AR was observed in 49% of patients and no AR was found in 39 patients (39%). Comparison of valve parameters before and after TAVI is shown in Table 2.

Out of 92 patients who were discharged from the hospital, 91 patients (98.9%) were alive at one-month follow-up. One patient expired due to non-cardiac reasons. NYHA class was significantly improved in patients as shown in Table 4. Out of the 92 patients, 13 patients (14.1%) got re-admitted in one month because of multiple issues.

**Table 4:** One-month follow-up NYHA, New York Heart Association; CCS, Canadian Cardiovascular Society.

Characteristics	Overall (n=92)
Alive status in one month	
Alive	91 [98.9%]
Death	1 [1.1%]
NYHA Functional Class after one month	
Class I	70 [76.1%]
Class II	21 [22.8%]
Unknown	1 [1.1%]
CCS angina status after one month	
No angina	47 [51.1%]
No limitation of physical activity	35 [38%]
Slight limitation of ordinary activity	10 [10.9%]

## Discussion

This study from Pakistan showed interesting results. The procedure was attempted for the first time in the region due to relatively newer concepts, cost constraints and procedure technicalities. The total number of cases done in the study period, were low. This is similar to Poland where in the start of the program, adaptation towards TAVI was low [14]. This is also consistent with the rest of Asian countries where overall adaptation for TAVI is low. Other reasons can be because of overall lower age of population compared with Europe and the United States, high cost of device, lack of screening facilities, unavailability of structured training program and presence of potentially challenging anatomical features i.e. bicuspid aortic valve [15,16].

Success rate in our study was consistent with success rate already reported in other studies [17]. In FRANCE 2 study, procedural success rate was 96.9% [18]. Slightly lower success rate in our study (94%) as compared to some other studies can be explained by the fact that we selected mainly moderate to high-risk patients. Majority were of functional class III and were refused for surgery. Our cohort also included high-risk post CABG patients. These results are incomparable with other large studies like PARTNER trials. As all of these studies didn’t include patients with bicuspid aortic valves [19,20]. Patients with bicuspid aortic stenosis have been generally excluded from prior TAVI trials due to asymmetric calcification and elliptical shape that may lead to under expansion of valve and para-valvular leak (PVL). There is also controversy about annular or supra =-annular measurement in bicuspid aortic valves. It is clear from international data that bicuspid aortic valve patients have higher risk and decreased success rate as compared with those with tricuspid aortic valve [21]. So another reason for lower success rate in our study as compared to other studies, is the inclusion of patients with bicuspid aortic valve involvement. Operator experience and developing institutional protocols may also be considered as a reason for lower success rate during our learning phase. It is seen that with newer generation valve, there was no increase in mortality although the PVL and permanent pacemaker implant was still increased [22].

Comparing with International data, successful valve deployment is similar. Seven valves were deployed ectopically so bailout with second valve. It is higher as compared to other studies which showed multiple valve implantation in only 2% of the cases [12]. Considering the initial and growing phase of TAVI program, ectopic valve deployment is expected. Of the possible reason for ectopic valve implantation could be the fact that the majority of our patients had bicuspid aortic valve which has less calcium so less valve holding capacity and the Core valve is not retrievable [23,24]. This is the reason that bicuspid valve has a higher need for the second valve (8%) [25].

The procedure-related mortality of TAVI in our study is similar to other studies but the overall in-hospital mortality of patients who underwent TAVI was higher. However, small sample size of the study may induce statistical bias and may lead to large variations in the results. If we compare our mortality data with other studies, there is a variability among different studies. Chakos et al. in their systemic review of 31 studies, which included 13,857 patients, reported 30-day mortality of TAVI as 8.4% [26]. Sehatzadeh et al. in their study showed that all-cause mortality in the first 30 days post TAVI was high, which was 5%, as compared to their SAVR group [12]. In SOURCE registry which included data from 32 European centers, their 30-day mortality was 6.3% [17]. A study in Germany showed mortality of 5.1% with trans-femoral TAVI [27].

Another observation found in our study was the learning curve regarding selection of equipment which played a major role in the reduction of the mortality in cases done later. Initially, Amplatz super stiff wire was used to deliver the valve and this led to catastrophic complications of left ventricular perforation. This made the operators to change practice and use Confida wire which has a softer tip. Similarly, two patients died of right ventricular perforation because of stiffer 6F TPM lead. This led to a change in practice and later TPM lead with balloon tip was used. This leads to a reduction in complications like perforation.

Higher incidence of AR was found in our study which is not surprising, as already different studies have reported that the use of Core valve, because of its manufacturing properties, is associated with more AR. It compelled the manufacturer to develop a newer version of Evolute-R and Evolute-R Pro. These newer versions are developed with a longer (Evolute -R) and extra Skurt (Evolute -R Pro) to minimize this complication [28]. After the procedure, when aortic regurgitation was measured through TTE it was comparable to international data in which Tarantini et al. showed that in 70% cases of TAVI, post TAVI mild AR was present [29]. Another reason for increased level of AR was bicuspid aortic valve (due to its anatomical properties, elliptical annulus, valve positioning and opposition was challenging) hence leading to AR.

Different arrhythmias were observed in our cases. Among these arrhythmias, AV block was more common but usually transient. Twenty-two patients (22%) developed persistent complete heart block so permanent pacemaker implanted. This is much lower than the international statistics. According to SOURCE registry, incidence of complete heart block and subsequent permanent pacemaker implantation was needed in 26% of Core Valve [17]. Adams et al. in their study showed that permanent pacemaker implantation was higher in core-valve as compared to SAVR or Sapien valve TAVI [25, 30]. Most of our patients received Core-valve and in comparison with the data above, the rate of permanent pacing is low in our patients. Core valve, because of its Sub-annular extension, is more prone to develop AV blocks. These AV blocks are also more common with Ectopic valves and with sub-annular calcification. As we did not exclude sub-annular calcification and bicuspid aortic valves in our study, this can be a reason for more AV blocks in our patients.

Sehatzadeh et al. didn’t show any significant difference between readmission of TAVR and SAVR patients in their study. As compared to their readmission rate of 7.7% [12], our readmission rate was higher (14.1%) probably because of low threshold for readmission after this expensive procedure. Patients got readmitted even for non-cardiac issues because of TAVI being a relatively newer procedure and lack of other hospitals to have experience in managing post-TAVI patients.

One-month follow-up of our patients showed that there was a significant improvement in NYHA functional class. This is consistent with PARTNER A [25] and US Core Valve Trial [30]. Small sample size and longer duration of the study were the main limitations of the study. As there are other centers in this part of the world who are doing TAVI, the power of the study can be improved by incorporating with them and having multicenter study. 

With advancement in the use of CT, increasing operator experience, improved patient selection, advanced device delivery system and newer-generation valves, TAVI is advancing. O’Sullivan anticipated that by 2020, majority of patients worldwide will undergo TAVI as first-line of management [22]. So with all the above features of the initial experience of TAVI program in NICVD, it is mandatory to have this new and growing mode of treatment in the country. With the passage of time, increasing operator experiences and learning from this exercise, we expect that the TAVI program will further improve and fulfil the requirement of this developing region.

## Conclusions

This study in a developing country like Pakistan may be a paradigm-shifting study. It showed that TAVI is safe and effective alternate to surgical aortic valve replacement in Pakistan in high-risk severe AS patients. After TAVI, there was a significant improvement in the quality of life (functional class) in those patients who were otherwise left on medical management because of high surgical risk. This new procedure will help in opening new hope and non-surgical option for patients who were otherwise left untreated. 
